# Power and sample size determination for the group comparison of patient-reported outcomes using the Rasch model: impact of a misspecification of the parameters

**DOI:** 10.1186/s12874-015-0011-4

**Published:** 2015-03-15

**Authors:** Myriam Blanchin, Alice Guilleux, Bastien Perrot, Angélique Bonnaud-Antignac, Jean-Benoit Hardouin, Véronique Sébille

**Affiliations:** EA 4275, Biostatistics, Pharmacoepidemiology and Subjective Measures in Health Sciences, University of Nantes, 1 rue, Gaston Veil, 44000 Nantes France

**Keywords:** Rasch model, Sample size, Power, Group comparison, Misspecification, Variance, Item parameters

## Abstract

**Background:**

Patient-reported outcomes (PRO) are important as endpoints in clinical trials and epidemiological studies. Guidelines for the development of PRO instruments and analysis of PRO data have emphasized the need to report methods used for sample size planning. The Raschpower procedure has been proposed for sample size and power determination for the comparison of PROs in cross-sectional studies comparing two groups of patients when an item reponse model, the Rasch model, is intended to be used for analysis. The power determination of the test of the group effect using Raschpower requires several parameters to be fixed at the planning stage including the item parameters and the variance of the latent variable. Wrong choices regarding these parameters can impact the expected power and the planned sample size to a greater or lesser extent depending on the magnitude of the erroneous assumptions.

**Methods:**

The impact of a misspecification of the variance of the latent variable or of the item parameters on the determination of the power using the Raschpower procedure was investigated through the comparison of the estimations of the power in different situations.

**Results:**

The power of the test of the group effect estimated with Raschpower remains stable or shows a very little decrease whatever the values of the item parameters. For most of the cases, the estimated power decreases when the variance of the latent trait increases. As a consequence, an underestimation of this variance will lead to an overestimation of the power of the group effect.

**Conclusion:**

A misspecification of the item difficulties regarding their overall pattern or their dispersion seems to have no or very little impact on the power of the test of the group effect. In contrast, a misspecification of the variance of the latent variable can have a strong impact as an underestimation of the variance will lead in some cases to an overestimation of the power at the design stage and may result in an underpowered study.

**Electronic supplementary material:**

The online version of this article (doi:10.1186/s12874-015-0011-4) contains supplementary material, which is available to authorized users.

## Background

Patient-reported outcomes (PRO) comprise a range of outcomes collected directly from the patient regarding the patient’s health, the disease and its treatment as well as their impact and include health related quality of life, satisfaction with care, psychological well-being... There has been growing interest in theses outcomes in the past years as they can be helpful to evaluate the effects of treatment on patient’s life or to study the quality of life of patient along with the disease progression to adapt the patient’s care [1-3]. The concept measured by PRO cannot be directly observed. In practice, patient-reported outcomes are assessed through questionnaires composed of items that indirectly measure a latent variable which represents the concept of interest. Two theories exist for the analysis of the responses of patients to items. The models from the Classical Test Theory are based on a score that often sums the responses to the items. Another theory has gained importance in patient-reported outcomes area [4], the Item Response Theory (IRT) including models which link the probability of a given answer to an item with item parameters and the latent variable. IRT has shown advantages such as the management of missing data [5], the possibility to obtain an interval measure for the latent trait, the comparison of latent traits levels independently of the instrument, the management of possible floor and ceiling effects [6,7].

Guidelines for the development of PRO instruments and analysis of PRO data have been developed [8-10] and have emphasized the need to report methods used for sample size planning. Indeed, sample size determination is essential at the design stage to achieve the desired power for detecting a clinically meaningful difference in the future analysis. An inadequate sample size may lead to misleading results and incorrect conclusions. Whereas an underestimated sample size may produce an underpowered study, an overestimated sample size raises ethical issues. A too large sample size will result in more included patients as would have been required, a longer follow-up period and a delayed analysis stage. All these problems may slow down the conclusion of the study and, for example, may delay an improvement of the medical care or the availability of a more efficient treatment towards patients.

The widely-used sample size formula for the comparison of two normally distributed endpoints in two independent groups of patients is based on a t-test. It has been recently highlighted that this formula was inadequate in the IRT setting [11]. In randomized clinical trials, Holman et al. [12] have first studied the power of the test of group effect for the two-parameter logistic model from the IRT. This simulation study investigated the power for various values of sample size, number of items and effect size in the context of a comparison of two groups answering a questionnaire composed of dichotomous items. This study was further extended [13] to compare different estimation methods of the power for the comparison of two groups in the context of dichotomously or polytomously scored items, and cross-sectional or longitudinal studies. These two simulation studies were based on the two-parameter logistic model from the IRT and its version for polytomous items, the generalized partial credit-model. In the framework of the Rasch model [14,15], Hardouin et al. [16] have proposed a methodology to determine the power of the Wald test of group effect for PRO cross-sectional studies comparing two groups of patients named the Raschpower procedure. In order to validate this theoretical approach, the power computed using Raschpower was compared to the power obtained in several simulation studies corresponding to different cases (cross-sectional [16,17] and longitudinal studies [18], well or misspecified Rasch models [19]). As the Raschpower procedure strongly relies on the mixed Rasch model that assumes the normality of the distribution of the latent variable, the robustness of this procedure to violation of the underlying model assumptions was also assessed [20]. The power obtained with the Raschpower method assuming normal distribution were compared to reference power obtained from data simulated with a non-normal distribution (a beta distribution leading to U, L or J-shaped distributions). Simulation studies have shown that the powers of group effect obtained either from the Raschpower procedure or from the simulated datasets were close to each other. In conclusion, the Raschpower procedure seems robust to non-normality of the latent variable.

The power determination using Raschpower in cross-sectional studies depends on the expected values of the following parameters: the sample size in each group, the number of items, the group effect defined as the expected difference between the means of the latent trait of each group, the item parameters and the variance of the latent trait. These expected values are required at the design stage and it can turn out to be problematic if no previous studies can provide some information on their values. If the expected values at the design stage are far from the estimated values in the study at the analysis stage, the power for a determined sample size could then not be achieved. As the variance of the latent trait and the item parameters are difficult to set at the planning stage of a study, it is highly probable that their expected values will be different from the observed values at the analysis stage. Therefore, the power of the study might be different from the expected power to a greater or lesser extent depending on the magnitude of the erroneous assumptions regarding the value of all the parameters of the study. The objective of this work is to study the impact of a misspecification of the variance of the latent variable or of the item parameters on the determination of the power using the Raschpower procedure.

## Methods

### Sample size and power determinations using the Rasch model

#### The latent regression Rasch model

The Rasch model [14,15] coming from the Item Response Theory models is a largely used model for dichotomous items. In this model, the link between the probability of an answer to an item and a latent variable (the non-directly observable variable that the PRO instrument intends to measure) as well as item difficulties is modeled. The probability that a person *i* answers a response *x*_*ij*_ to an item *j* is expressed by a logistic model with two parameters, (i) the value of the latent variable of the person, *θ*_*i*_ and (ii) the difficulty of the item *j*, *δ*_*j*_. For a questionnaire composed of *J* dichotomous items answered by *N* patients, the mixed Rasch model can be written as follows:

(1) $$ \begin{aligned} Pr\left(X_{ij}=x_{ij}|\theta, \delta_{j}\right)&=\frac{exp\left(x_{ij}\left(\theta-\delta_{j}\right)\right)}{1+exp\left(\theta-\delta_{j}\right)} \\ i&=1,...,N, j=1,...,J \\ \Theta & \sim N\left(\mu,\sigma_{\theta}^{2}\right) \end{aligned}  $$

where *x*_*ij*_ is a realization of the random variable *X*_*ij*_. *θ* is a realization of the random variable *Θ*, generally assumed to have a gaussian distribution. The parameters of the Rasch model can then be estimated by marginal maximum likelihood (MML) [21]. A constraint has to be adopted to ensure the identifiability of the model: the mean of the latent variable is often constrained to 0 (*μ*=0).

In the context of a study comparing two groups of patients, the latent regression Rasch model also estimates the group effect, *γ*, defined as the difference between the means of the latent variable in the two groups (*μ*_0_ for group 0 and *μ*_1_ for group 1). The latent regression Rasch model can be written as follows:

(2) $$ \begin{aligned} Pr\left(X_{ij}=x_{ij}|\theta, \delta_{j}, \gamma\right)&=\frac{exp\left(x_{ij}\left(\theta+g_{i}\gamma-\delta_{j}\right)\right)}{1+exp\left(\theta+g_{i}\gamma-\delta_{j}\right)} \\ \Theta & \sim N(\mu,\sigma_{\theta}^{2}) \end{aligned}  $$

The mean of the latent variable *μ* is defined as the mean between *μ*_0_ and *μ*_1_, each of them weighted by the sample sizes *N*_0_ for group 0 and *N*_1_ for group 1. Consequently,

$$\left\{ \begin{aligned} \mu=N_{0}\mu_{0}+N_{1}\mu_{1}=0 \\ \gamma=\mu_{1}-\mu_{0} \end{aligned} \right. \Longleftrightarrow \left\{ \begin{aligned} \mu_{0}=-\frac{N_{1}\gamma}{N_{0}+N_{1}} \\ \mu_{1}=\frac{N_{0}\gamma}{N_{0}+N_{1}} \end{aligned} \right. $$

As a consequence, $g_{i}=-\frac {N_{1}}{N_{0}+N_{1}}$ for individuals in the group 0 and $g_{i}=\frac {N_{0}}{N_{0}+N_{1}}$ for individuals in the group 1 in order to meet the constraint of identifiability, *μ*=0.

#### The Raschpower procedure for power estimation

The Raschpower procedure provides an estimation of the power for the comparison of PRO data in two independent groups of patients when a Rasch family model is intended to be used for the analysis. This procedure is used at the planning stage and is based on a Wald test to detect a group effect. To perform the test of group effect, an estimate *Γ* of the group effect *γ* and its standard error are required. Since no dataset exists during the planning stage, no estimate can be obtained from data. Hardouin et al. [16] proposed to obtain a numerical estimation for the standard error of *Γ* from an expected dataset of the patients’ responses.

In this procedure, a dataset of the patients’ responses is first created conditionally on the planning expected values for the sample size in each group (*N*_0_ and *N*_1_), the group effect (*γ*), the item difficulties (*δ*_*j*_) and the variance of the latent trait $\left (\sigma _{\theta }^{2}\right)$. All possible response patterns of the patients are determined. The associated probability and the expected frequency of each response pattern for each group are computed using the mixed Rasch model (eq. 1) given the planning expected values. The expected dataset is composed of all possible response patterns and their associated frequencies.

Then, to estimate the standard error of the group effect, the expected dataset is analysed with a latent regression mixed Rasch model (eq. 2) where the item difficulties *δ*_*j*_ and the variance of the latent trait $\sigma _{\theta }^{2}$ are set to the planning expected values. The Wald test of group effect is performed with the hypotheses *H*_0_:*γ*=0 against *H*_1_:*γ*≠0 and the test statistic $\frac {\Gamma }{\sqrt {var(\Gamma)}}$ follows a standard normal distribution under *H*_0_. The expected power of the test of the group effect, $1-\hat {\beta }$, can be approximated by [16]:

(3) $$ 1-\hat{\beta}\approx1-\Phi\left(z_{1-\alpha/2}-\frac{\gamma}{\sqrt{\hat{var}(\hat\gamma)}}\right)  $$

with *γ* assumed to take on positive values, *z*_1−*α*/2_ be the quantile of the standard normal distribution and $\hat {var}\left (\hat \gamma \right)$ estimated from the expected dataset. In order to validate the Raschpower procedure, the power computed using Raschpower was compared previously to the power obtained in several simulation studies. The following parameters could vary in the simulation studies: the sample size (in each group or at each time, *N*=50, 100, 200, 300, 500), the group or time effect (*γ*=0.2,0.5,0.8), the variance of the latent traits $({\sigma _{1}^{2}}={\sigma _{2}^{2}}=0.25, 1, 4, 9$), the correlation of the latent traits between two times of measurement for longitudinal studies (*ρ*=0.4, 0.7, 0.9), the number of items (*J*=5 or 10) and of response categories (*K*=2, 3, 5, 7). In this study, a large set of values of the variance of the latent variable and item parameters are examined to evaluate the impact of a misspecification of these parameters.

### Misspecification of the variance of the latent variable

To determine the impact of a misspecification of the variance of the latent variable, we have compared different estimations of the power estimated with Raschpower for a large set of values of $\sigma _{\theta }^{2}=\{0.25,0.5,0.75$, 1,1.5,2,2.5,3,4,5,6,7,8,9}. By comparing the estimations of the power, the impact of an under/overestimation of the variance at the planning stage can be assessed. All the parameters used at the planning stage could vary: the sample size in each group (*N*_0_=*N*_1_=50,100,200,300,500), the number of items (*J*=3,5,7,9,11,13,15), the value of the group effect (*γ*=0.1,0.2,0.5,0.8). The item difficulties were drawn from the percentiles of a normal distribution with the same characteristics as the latent variable distribution $N\left (0,\sigma _{\theta }^{2}\right)$.

### Misspecification of the item difficulties

To determine the impact of a misspecification of the item difficulties, we have compared the power estimated with Raschpower for a large set of values of *δ*_*j*_. The item difficulties were drawn from the percentiles of the item distribution defined as an equiprobable mixture of two normal distributions *N*(−*a*;0.1*x*^2^) and *N*(*a*;*x*^2^) where *a* is the gap between the means of the two normal distributions. As a consequence, the mean of the item distribution is equal to 0 and $x^{2}=\left (\sigma ^{2}_{\delta _{j}}-a^{2}\right)/0.55$ can be expressed as a function of *a* and $\sigma ^{2}_{\delta _{j}}$, the variance of the item distribution. The equiprobable mixture for generating item distribution easily creates two types of distribution: unimodal and bimodal. A unimodal distribution of the item difficulties reflects the situation where the questionnaire is perfectly suitable for a population with normally distributed latent traits, which is the case here, contrary to a bimodal distribution. The equiprobable mixture also creates a large number of item distributions in which item difficulties can be more or less regularly spaced which may impact the results of Raschpower.

**Figure 1:**
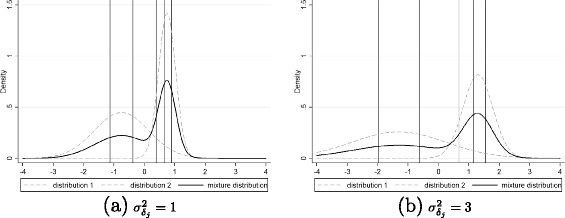
**Density of mixture distribution for** $J=5, a=-0{.}75, \sigma ^{2}_{\theta }=1$ **and different values of** $\sigma ^{2}_{\delta _{j}}$ **.** Vertical lines represent the values of the item difficulties drawn from the mixture distribution. Item difficulties for $\sigma ^{2}_{\delta _{j}}=1$ (Figure **a**): *δ*_*j*_=(−1.13,−0.37,0.39,0.67,0.9). Item difficulties for $\sigma ^{2}_{\delta _{j}}=3$ (Figure **b**): *δ*_*j*_=(−1.96,−0.63,0.68,1.16,1.55).

**Figure 2:**
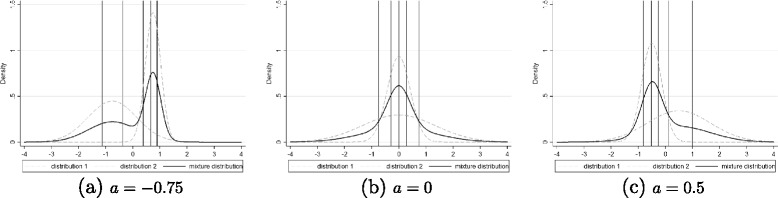
**Density of mixture distribution for** $J=5, \sigma ^{2}_{\theta }=1, \sigma ^{2}_{\delta _{j}}=1$ **and different values of** ***a*** **.** Vertical lines represent the values of the item difficulties drawn from the mixture distribution. Item difficulties for *a*=−0.75 (Figure **a**): *δ*_*j*_=(−1.13,−0.37,0.39,0.67,0.9). Item difficulties for *a*=0 (Figure **b**): *δ*_*j*_=(−0.74,−0.29,0,0.29,0.74). Item difficulties for *a*=0.5 (Figure **c**): *δ*_*j*_=(−0.81,−0.52,−0.26,0.13,1).

**Figure 3:**
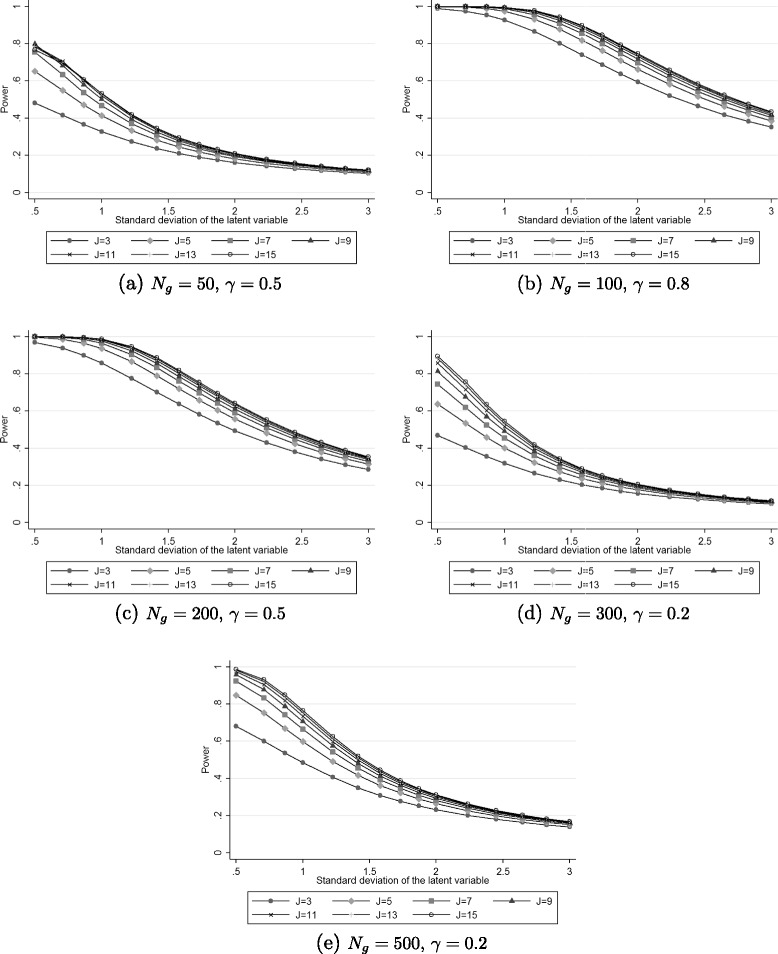
**Power estimated with Raschpower as a function of the standard deviation of the latent variable and the number of items (** ***J*** **) for 50 patients per group and a group effect=0.5 (Figure a), 100 patients per group and a group effect=0.8 (Figure b), 200 patients per group and a group effect=0.5 (Figure c), for 300 patients per group and a group effect=0.2 (Figure d) or 500 patients per group and a group effect=0.2 (Figure e).**

The misspecification of the item difficulties was created using the variation of $\sigma ^{2}_{\delta _{j}}=\{0.25,0.5,0.75,1$, 1.5,2,2.5,3,4,5,6,7,8,9} and of the gap between the means of the two normal distributions $a=\left \{-3/4\sigma _{\delta _{j}},\, -1/2\sigma _{\delta _{j}}, \, -1/4\sigma _{\delta _{j}},\, 0, 1/4\sigma _{\delta _{j}},\,1/2\sigma _{\delta _{j}},\,3/4\sigma _{\delta _{j}}\right \}$. A variation in the variance of the item distribution implies a variation of the intervals between the values of the item difficulties drawn from this distribution. An example of the effect of a variation of $\sigma ^{2}_{\delta _{j}}$ when *J*=5 and $\sigma ^{2}_{\theta }=1$ is represented in Figure 1. If the variance $\sigma ^{2}_{\delta _{j}}$ increases, e.g. from 1 in Figure 1(a) to 3 in Figure 1(b), the intervals between item difficulties increase. As a consequence, the easiest items become easier and the most difficult items become more difficult. An increase of the variance creates a shift of the item difficulties at both ends of the item difficulties distribution without changing the overall pattern. However, a variation of the gap between the means *a* leads to changing the overall pattern of the item difficulties as it alters the shape of the equiprobable mixture distribution as shown in Figure 2. We can note that for *a*=0 such as in Figure 2(b), a unimodal distribution is obtained and the item difficulties are almost regularly spaced. When *a*<0 such as in Figure 2(a), items difficulties on the left of the distribution are more spaced than item difficulties on the right and so the estimations of the latent variable will be more accurate on the right, and inversely when *a*>0 such as in Figure 2(c). Furthermore, to avoid ceiling and floor effects and ensure that the questionnaire was suitable for the population (not too specific nor too generic) [17], we decided to exclude cases where $\sigma ^{2}_{\delta _{j}}>$$8 \times \sigma _{\theta }^{2}$ with $\sigma _{\theta }^{2}=\{0.25,0.5,0.75,1,1.5,2,2.5,3,4,5,6$, 7,8,9}. The other parameters used at the planning stage could also vary: the sample size in each group (*N*_0_=*N*_1_=50,100,200,300,500), the number of items (*J*=3,5,7,9,11,13,15), the value of the group effect (*γ*=0.1,0.2,0.5,0.8).

The draw of the parameters and the estimation of power using the Raschpower procedure for all combinations of parameters were performed with Stata software.

## Results

### Misspecification of the variance of the latent variable

Table 1 shows the power estimated with Raschpower for some values of the variance of the latent variable $\left (\sigma ^{2}_{\theta }\right)$, the number of items (*J*), the group effect (*γ*) and the sample size per group (*N*_*g*_). The results for all values of the parameters are presented in Additional file 1. As expected, the estimated power increases with the number of items, the group effect and the sample size. For most of the cases as represented in Figure 3(a), (d) and (e), the estimated power decreases when the variance of the latent trait increases. As a consequence, an underestimation of the variance $\sigma ^{2}_{\theta }$ will lead to an overestimation of the power at the design stage and finally to an underpowered study. The loss of power, corresponding to the decrease between the expected power and the observed power, due to an underestimation of the variance is the highest for small values of the variance $\sigma ^{2}_{\theta }$ and high values of *J*. For example, for *J*=15, *N*_*g*_=300 and *γ*=0.2, the power is estimated at 89.5% for $\sigma ^{2}_{\theta }=0.25$ and at 75.7% for $\sigma ^{2}_{\theta }=0.5$. So, an underestimation of 0.25 of the variance of the latent variable in this example leads to a decrease of 13.8% of the power of the test of group effect. On the opposite, the power is estimated at 20.6% for $\sigma ^{2}_{\theta }=4$ and at 17.6% for $\sigma ^{2}_{\theta }=5$ under the same conditions. Therefore, an underestimation of 1 of the variance of the latent variable in this case leads to a decrease of power of only 3.0%.

**Table 1:** **Power estimated with the Raschpower procedure for different values of the variance of the latent variable (** $\sigma ^{2}_{\theta }$ **), the number of items (** ***J*** **), the group effect (** ***γ*** **) and the sample size per group (** ***N***_***g***_ **)**

***J***	***N***_g_	***γ***	${\sigma ^{2}_{\theta }=0.25}$	${\sigma ^{2}_{\theta }=0.5}$	${\sigma ^{2}_{\theta }=0.75}$	${\sigma ^{2}_{\theta }=1}$	${\sigma ^{2}_{\theta }=2}$	${\sigma ^{2}_{\theta }=4}$	${\sigma ^{2}_{\theta }=9}$
3	50	0.1	0.058	0.054	0.051	0.049	0.044	0.039	0.034
		0.2	0.117	0.104	0.095	0.088	0.072	0.058	0.046
		0.5	0.482	0.417	0.367	0.328	0.237	0.162	0.104
		0.8	0.859	0.793	0.731	0.677	0.511	0.343	0.199
3	200	0.1	0.117	0.104	0.095	0.088	0.072	0.058	0.046
		0.2	0.337	0.289	0.254	0.229	0.168	0.119	0.081
		0.5	0.969	0.938	0.900	0.859	0.702	0.495	0.287
		0.8	1.000	1.000	0.999	0.998	0.978	0.875	0.607
3	500	0.1	0.229	0.198	0.176	0.159	0.121	0.090	0.064
		0.2	0.682	0.602	0.538	0.485	0.351	0.234	0.141
		0.5	1.000	1.000	0.999	0.998	0.976	0.868	0.598
		0.8	1.000	1.000	1.000	1.000	1.000	0.998	0.942
9	50	0.1	0.084	0.071	0.064	0.059	0.049	0.041	0.036
		0.2	0.209	0.164	0.138	0.121	0.088	0.065	0.049
		0.5	0.798	0.682	0.579	0.501	0.325	0.200	0.118
		0.8	0.991	0.970	0.929	0.877	0.674	0.433	0.234
9	200	0.1	0.213	0.165	0.138	0.121	0.088	0.065	0.049
		0.2	0.643	0.505	0.415	0.352	0.227	0.144	0.090
		0.5	1.000	0.998	0.992	0.977	0.856	0.612	0.340
		0.8	1.000	1.000	1.000	1.000	0.998	0.948	0.696
9	500	0.1	0.453	0.345	0.281	0.239	0.158	0.106	0.071
		0.2	0.958	0.878	0.788	0.707	0.482	0.295	0.163
		0.5	1.000	1.000	1.000	1.000	0.998	0.944	0.686
		0.8	1.000	1.000	1.000	1.000	1.000	1.000	0.974
15	50	0.1	0.094	0.077	0.067	0.061	0.050	0.042	0.036
		0.2	0.228	0.181	0.149	0.129	0.091	0.067	0.050
		0.5	0.768	0.695	0.607	0.532	0.346	0.210	0.122
		0.8	0.989	0.962	0.932	0.895	0.703	0.455	0.244
15	200	0.1	0.263	0.190	0.154	0.132	0.092	0.067	0.050
		0.2	0.737	0.578	0.467	0.392	0.245	0.152	0.093
		0.5	1.000	1.000	0.996	0.987	0.887	0.642	0.355
		0.8	1.000	1.000	1.000	1.000	0.999	0.961	0.719
15	500	0.1	0.562	0.408	0.322	0.269	0.170	0.111	0.072
		0.2	0.987	0.932	0.850	0.766	0.521	0.313	0.170
		0.5	1.000	1.000	1.000	1.000	0.999	0.957	0.709
		0.8	1.000	1.000	1.000	1.000	1.000	1.000	0.980

For other cases as represented in Figures 3(b) and 3(c), the estimated power first stays stable at 100% for small values of variance and then decreases when the variance of the latent trait increases. This effect was observed for high values of the group effect *γ*. The combination of a high group effect and a low variance produces a very high standardized effect that can always be detected whatever the values of the number of items and that explains the estimated power of 100%. In these cases, as soon as the power begins to decrease (for $\sigma ^{2}_{\theta }>1$ in Figure 3(c)), the same effects as before are observed i.e. an underestimation of the variance $\sigma ^{2}_{\theta }$ leads to a loss of power which is the highest for small values of the variance $\sigma ^{2}_{\theta }$ and high values of *J*.

### Misspecification of the item difficulties

**Figure 4:**
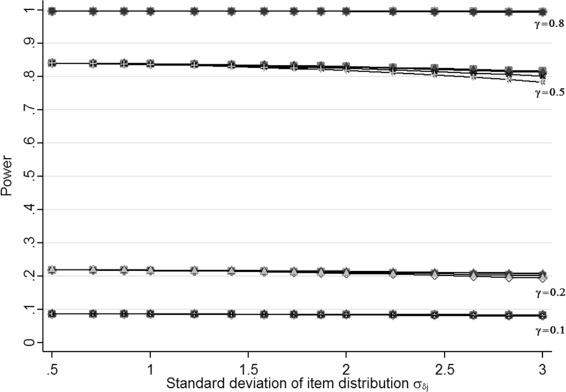
**Power estimated with Raschpower as a function of the standard deviation of the item distribution (** ${\sigma _{\delta _{j}}}$ **), the group effect (** ***γ*** **) and the gap between the means of the normal distributions (** ***a*** **) for a sample size per group** ***N***_***g***_ **=200, a number of items** ***J*** **=7 and a variance of the latent variable** ${\sigma ^{2}_{\theta }=2}$ **.** Overlaid curves represent different values of *a*, $a=\left \{-3/4\sigma _{\delta _{j}},-1/2\sigma _{\delta _{j}}, -1/4\sigma _{\delta _{j}}, 0, 1/4\sigma _{\delta _{j}},1/2\sigma _{\delta _{j}},3/4\sigma _{\delta _{j}}\right \}$.

Table 2 shows the power estimated with Raschpower for some values of the sample size per group (*N*_*g*_), the group effect (*γ*), the variance of the item distribution $\left (\sigma ^{2}_{\delta _{j}}\right)$ and the gap between the means of the two normal distributions (*a*) when the variance of the latent variable $\sigma ^{2}_{\theta }$=1 and the number of items *J*=7. The results for all the values of the sample size, the group effect, the variance of the item distribution and the gap between the means of the two normal distributions and values for the variance of the latent variable equals to 0.25, 0.5, 1, 2, 4 or 9 and for the number of item equals to 3, 9 or 15 respectively are presented in Additional file 2. The impact of a misspecification of the item difficulties was the same whatever the values of the number of items (*J*), the sample size per group (*N*_*g*_) and the variance of the latent trait $\left (\sigma ^{2}_{\theta }\right)$ (results not shown). In general, the estimated power remains stable or shows a very little decrease when the variance of the item distribution $\sigma ^{2}_{\delta _{j}}$ or the gap between the means of the two normal distributions *a* increases. It seems that a misspecification of the item difficulties regarding their overall pattern (change in *a*, Figure 2) or their dispersion (change in $\sigma ^{2}_{\delta _{j}}$, Figure 1) has no or very little impact on the power. In some extreme cases, where the gap between the means of the two normal distributions is high and the variance of the item distribution is high compared to the variance of the latent trait, a small decrease of the power is observed. An illustration of this effect is presented in Figure 4. We can observe that the power for *γ*=0.5 decreases when the variance of the item distribution increases and that the curves are no more overlaid for $\sigma ^{2}_{\delta _{j}}\geq 4$. In this case, the power decreases more for high values of *a*$\left (a=\pm 3/4\sigma _{\delta _{j}}\right)$. In fact, for *γ*=0.5, *N*_*g*_=200 and *J*=7 the power without misspecification $\left (a=0\, \text {and}\, \sigma ^{2}_{\delta _{j}}=\sigma ^{2}_{\theta }=2\right)$ is estimated at 83.5% whereas the power is estimated at 78.3% in case of a high misspecification $\left (a=\pm 3/4\sigma _{\delta _{j}}\, \text {and} \,\sigma ^{2}_{\delta _{j}}=9=4.5\times \sigma ^{2}_{\theta }\right)$ which results however in a decrease of power of only 5.2%.

**Table 2:** **Power estimated with the Raschpower procedure for different values of the sample size per group (** ***N***_***g***_ **), the group effect (** ***γ*** **), the variance of the item distribution** $\left (\sigma ^{2}_{\delta _{j}}\right)$ **and the gap between the means of the two normal distributions (** ***a*** **) when the variance of the latent variable** $\sigma ^{2}_{\theta }$ **=1 and the number of items** ***J*** **=7**

***N***_***g***_	***γ***	${\sigma ^{2}_{\delta _{j}}}$	***a*** **=0**	${a=\pm \frac {1}{4}\sigma _{\delta _{j}}}$	${a=\pm \frac {1}{2}\sigma _{\delta _{j}}}$	${a=\pm \frac {3}{4}\sigma _{\delta _{j}}}$
50	0.1	0.25	0.057	0.057	0.057	0.057
		1	0.057	0.057	0.057	0.057
		8	0.055	0.054	0.053	0.052
50	0.2	0.25	0.115	0.115	0.115	0.115
		1	0.114	0.114	0.114	0.113
		8	0.107	0.106	0.103	0.099
50	0.5	0.25	0.475	0.474	0.474	0.473
		1	0.472	0.471	0.469	0.466
		8	0.432	0.427	0.413	0.387
50	0.8	0.25	0.854	0.855	0.856	0.855
		1	0.852	0.852	0.850	0.848
		8	0.815	0.810	0.794	0.764
200	0.1	0.25	0.116	0.116	0.116	0.116
		1	0.115	0.115	0.114	0.114
		8	0.107	0.106	0.103	0.099
200	0.2	0.25	0.333	0.332	0.332	0.331
		1	0.329	0.328	0.326	0.324
		8	0.299	0.296	0.286	0.268
200	0.5	0.25	0.968	0.968	0.968	0.967
		1	0.966	0.966	0.965	0.964
		8	0.947	0.945	0.936	0.918
200	0.8	0.25	1.000	1.000	1.000	1.000
		1	1.000	1.000	1.000	1.000
		8	1.000	1.000	1.000	1.000
500	0.1	0.25	0.226	0.226	0.226	0.225
		1	0.223	0.223	0.222	0.220
		8	0.204	0.202	0.195	0.184
500	0.2	0.25	0.675	0.675	0.674	0.673
		1	0.669	0.668	0.666	0.662
		8	0.621	0.615	0.596	0.563
500	0.5	0.25	1.000	1.000	1.000	1.000
		1	1.000	1.000	1.000	1.000
		8	1.000	1.000	1.000	1.000
500	0.8	0.25	1.000	1.000	1.000	1.000
		1	1.000	1.000	1.000	1.000
		8	1.000	1.000	1.000	1.000

## Illustrative example

The ELCCA (Etude Longitudinale des Changements psycho-économiques liés au CAncer) study is a longitudinal prospective study that enrolled breast cancer and melanoma patients and was approved by an ethical research committee (CPP) prior to being carried out in the department of onco-dermatology at Nantes University Hospital (for melanoma patients) and at Nantes Institut de Cancérologie de l’Ouest (for breast cancer patients). This study aimed at analyzing the evolution of the life satisfaction (Satisfaction With Life Scale) of patients after cancer and its interaction with the health-related quality of life (EORTC QLQ-C30), the economic situation and the disease-related psychological changes (Post-Traumatic Growth Inventory [22]) measured at different times (1, 6, 12 and 24 months after diagnosis). Positive changes after cancer experience have been highlighted in several studies on the post traumatic growth, especially regarding life priorities and relation with the others. The impact of a misspecification of the parameters on the power determination can be illustrated by to determining the *a priori* power of the test of group effect between breast cancer and melanoma patients regarding the dimension “relation with others” of the post-traumatic growth inventory in the ELCCA study at 6 months post-diagnosis (first period of change). The dimension “relation with others” is composed of 7 items having 6 response categories. To determine the power, the Raschpower procedure required the expected values of the following parameters: (i) the group effect, (ii) the number of items: *J*=7, (iii) the item parameters, (iv) the variance of the latent variable and (v) the sample size in each group (*n*_0_=213 for breast cancer and *n*_1_=78 for melanoma). The choice of expected values for these parameters may be tough and can be guided by a pilot study.

The determination of the *a priori* power of the ELCCA study can rely on the estimated group effect, item parameters and variance from a pilot study including 20 breast cancer patients and 10 melanoma patients from a subsample of the ELCCA study at 6 months post-diagnosis. The parameters are estimated from a partial-credit model. They have the advantage to come from a similar population but are face with a lack of accuracy due to the small sample size. On these 30 patients, the estimations were $\hat {\gamma }_{\textit {PILOT}}=0.1888$ (standard error=0.310) for the group effect, $\hat {\sigma }_{\textit {PILOT}}^{2}=0.7858$ (standard error=0.321) for the variance of the latent variable and $\hat {\delta }_{\textit {jpPILOT}}=\left (\begin {array}{ccccc} -1.7611 & -0.3218 & -1.1222 & -0.5098 & 3.1989\\ -0.2020 & -1.4045 & -0.9434 & 0.7206 & 2.7450\\ -0.8376 & 0.3936 & -1.1745 & 1.3828 & 2.3384\\ -0.6028 & -0.5042 & -1.3033 & 0.1700 & 2.1788\\ -1.3974 & -0.2322 & -1.1994 & 1.2214 & 2.4814\\ -0.0203 & -1.0380 & -0.5060 & 1.1662 &. \\ -2.2906 & 1.6257 & -2.7366 & 1.8800 & 1.2561 \end {array}\right)$for the item parameters. We can note that the standard errors of the parameters are large. Moreover, it appears that nobody has chosen the last response category of the 6th item and consequently, the corresponding item parameter is missing. This value is required to perform the Raschpower procedure and we choose to linearly extrapolate the item parameter of the last response category from the two previous one of the same item. So, the missing item parameter is replaced by 1.1662+(1.1662–0.5060)=2.8384. With these estimated parameters, the *a priori* power determined with Raschpower is equal to 38.37% as shown in Table 3.

**Table 3:** ***A priori*** **power estimated with the Raschpower procedure from a pilot study and impact of misspecified parameters on the power (** $1-\hat {\beta }$ **)**

**Estimations used to determine the power with Raschpower**			**Estimated power**	**Misspecified parameters**	
**Item parameters**	**Group effect**	**Variance of the latent variable**	${1-\hat {\beta }}$	**Item parameters**	**Variance**
Pilot: $\hat {\delta }_{\textit {jpPILOT}}$	$\hat {\gamma }_{\textit {PILOT}}=0.1888$	Pilot: $\hat {\sigma }_{\textit {PILOT}}^{2}=0.7858$	0.3837 (*a priori*)		
ELCCA: $\hat {\delta }_{\textit {jpELCCA}}$	$\hat {\gamma }_{\textit {PILOT}}=0.1888$	Pilot: $\hat {\sigma }_{\textit {PILOT}}^{2}=0.7858$	0.3771	YES	
Pilot: $\hat {\delta }_{\textit {jpPILOT}}$	$\hat {\gamma }_{\textit {PILOT}}=0.1888$	ELCCA: $\hat {\sigma }_{\textit {ELCCA}}^{2}=1.0864$	0.3004		YES
ELCCA: $\hat {\delta }_{\textit {jpELCCA}}$	$\hat {\gamma }_{\textit {PILOT}}=0.1888$	ELCCA: $\hat {\sigma }_{\textit {ELCCA}}^{2}=1.0864$	0.2983	YES	YES

Since the ELCCA data have been collected, we can now look at the estimations of the item parameters of the ELCCA study, $\hat {\delta }_{\textit {jpELCCA}}=\left (\begin {array}{ccccc} -0.9735 & -1.0501 & -1.7684 & -0.0987 & 2.1514\\[.5pt] -0.6494 & -0.9946 & -1.3959 & 0.7675 & 2.1610\\[.5pt] -0.2551 & -0.9686 & -0.9510 & 1.3100 & 2.4691\\[.5pt] -0.3091 & -1.2309 & -1.6587 & 0.5290 & 2.1048\\[.5pt] -0.5618 & -1.4289 & -1.2661 & 1.1758 & 2.5396\\[.5pt] -0.6131 & -1.2691 & -1.5260 & 1.0783 & 2.3768\\[.5pt] -0.9466 & -0.5453 & -1.9003 & 1.0190 & 2.7882 \end {array}\right)$. As the final item parameters estimated from ELCCA are noticeably different from the item parameters estimated from the pilot study used to determine the *a priori* power, we can wonder how much the power is impacted by this misspecification of the item parameters. The power determined with the final item parameters estimated from ELCCA (line 2 of Table 3) and the group effect and the variance estimated from the pilot study is equal to 37.71%. So, using the item parameters from the pilot study has led to underestimate the power by around 1%. Similarly, we can look at the estimated variance of the latent variable in ELCCA, $\hat {\sigma }_{\textit {ELCCA}}^{2}=1.0864$. The power determined with the final variance estimated from ELCCA (line 3 of Table 3) is equal to 30.04%. So, the underestimation of the variance (0.79 instead of 1.09) has led to overestimate the power by 8%. If we now look at the combined effect of misspecifying the item parameters and the variance, the power determined with the final item parameters and variance estimated from ELCCA (line 4 of Table 3) is equal to 29.83% and is not so far from the power where only the variance was misspecified (30.04%). It is clear from this example that the misspecification of the variance of the latent variable can have a large impact on the determination of the power whereas a misspecification of the item parameters has less impact.

Eventually, the *post hoc* power determined with the final group effect (${\hat {\gamma }_{\textit {ELCCA}}=-0.0408}$), variance ${(\hat {\sigma }_{\textit {ELCCA}}^{2}= 1.0864)}$ and item parameters ($\hat {\delta }_{\textit {jpELCCA}}$) estimated from the ELCCA study happens to be really small (1.15%) as the group effect is near 0.

## Discussion

The determination of the power of the test of group effect using Raschpower at the design stage relies on the planning expected values for the sample size in each group (*N*_0_ and *N*_1_), the group effect (*γ*), the item difficulties (*δ*_*j*_) and the variance of the latent trait $\left (\sigma _{\theta }^{2}\right)$. In this study, the impact of a misspecification of the item difficulties or the variance of the latent trait on the power was assessed through the comparison of the estimations of the power in different situations. It seems that a misspecification of the item difficulties regarding their overall pattern (change in *a*) or their dispersion (change in $\left.\sigma ^{2}_{\delta _{j}}\right)$ has no or very little impact on the power. The parameters *a* and $\sigma ^{2}_{\delta _{j}}$ characterize the equiprobable mixture of normal distributions from which the item difficulties were drawn. Their values were deliberately chosen to avoid ceiling and floor effects as the Raschpower procedure has been validated in previous work on cases where no or little ceiling and floor effects [17] are observed (when the mean of the latent variable is different from the mean of the item distribution, for similar variances). That’s why, in this study, the means of the latent variable and item distributions were equal and the different values of the variance of the item distribution $\sigma ^{2}_{\delta _{j}}$ were limited to $8 \times \sigma ^{2}_{\theta }$. It comes out that a misspecification of the item difficulties at design stage matters little as long as no floor or ceiling effect has been created by the misspecification.

Other distributions might have been chosen to draw the item difficulties distribution. However, it seems that the form of the distribution has very little impact on the determination of power with the Raschpower procedure. In contrast, the occurrence of floor or ceiling effects may impact the determination of the power. These effects are due to a gap between the means of the latent variable distribution and the items distribution. When these two distributions are not overlaid, some items can be too difficult or too easy for the population. The floor or ceiling effects can also results from an item distribution more spread out than the latent variable distribution where the easy items will be too easy and the difficult items will be too difficult for the population. So, the characteristics of the distribution seem to have more impact on the correct determination of the power rather than the form of the distribution. Therefore, we can expect similar results if the item parameters were drawn from a distribution having a different form but the same characteristics than the equiprobable mixture of normal distributions where no ceiling or floor effects occur.

In contrast, a misspecification of the variance of the latent variable can have a strong impact as an underestimation of the variance $\sigma ^{2}_{\theta }$ will lead to an overestimation of the power at the design stage and may result in an underpowered study. The decrease of power between the expected power and the observed power due to an underestimation of the variance is the highest for small values of the variance $\sigma ^{2}_{\theta }$ and high values of *J*. The observed decrease of power is due to the assumption that the value of the group effect was correctly specified at the design stage and that the misspecification occurred only on the variance. As a matter of fact, the increase of the variance of the latent variable $\sigma ^{2}_{\theta }$ causes the increase of the estimated variance of the group effect $\hat {var}\left (\hat \gamma \right)$. Hence, as the estimation of the power (equation 3) includes the ratio $\frac {\gamma }{\sqrt {\hat {var}(\hat \gamma)}}$, an increase of $\sigma ^{2}_{\theta }$ leads to a decrease of this ratio and eventually to a decrease of power. Furthermore, the assumption of a correct specification of the group effect also explains the observed plateau of the power at 100% for small values of $\sigma ^{2}_{\theta }$ and high values of *γ* as the standardized effect $\frac {\gamma }{\sigma _{\theta }}$ to detect is large and greater than 1.

The increase of power with the number of items, the group effect and the sample size is consistent with previous works in item reponse theory [12,13]. The good performance of the Raschpower procedure illustrated in different settings [16,18] strengthens the previous finding that methods based on marginal maximum likelihood estimations and accounting for the unreliability of the latent outcome provides adequate power in item response theory [13]. This study emphasizes the potential strong impact of misspecifying the variance of the latent variable in power and sample size determinations for PRO cross-sectional studies comparing two groups of patients. This effect of the variance is certainly not limited to the power and sample size determinations in the Rasch model or even in item response theory but also probably pertains to the sample size calculation based on observed variables. It must be noted that the expected value of variance should be cautiously chosen to compute a sample size and plan a study and carefully estimated to determine a *post hoc* power.

Even though this study of the impact of the misspecification of the parameters pertains to the comparison of PRO data evaluated by dichotomous items in two independent groups of patients, the Raschpower procedure was also developed for polytomous items and/or longitudinal studies [18]. We can assume that, in such settings, a misspecification of the variance may also have an impact on the estimation of the power whereas this estimation may not suffer from a misspecification of the item parameters. For longitudinal studies, the impact of a misspecification of the parameters will not only depend on the value of the variance of the latent variable *σ*^2^ but also on the whole covariance matrix, i.e. on the variance of the latent variable at each measurement occasion and its correlation between measurement occasions. For questionnaires composed of polytomous items, this impact will depend on the number of items and also on the number of response categories of the items.

A number of software programs or websites are useful for power analysis and sample size calculation. Some specialized programs (G*POWER, PASS, NQuery Advisor, PC-Size, PS) and some more general statistical programs (SAS, Stata, R) can provide power and sample size through the t-test based formula for the comparison of two normally distributed endpoints in two independent groups of patients. Unfortunately, this formula is not adequate in the Rasch model setting [11] and to our knowledge, the correct determination of the sample size or power for a study intended to be analysed with a Rasch model is not available on any softwares or websites. To provide an easy way to determine the sample size and power in this setting, the whole Raschpower procedure has been implemented in the Raschpower module freely available at the website PRO-online http://pro-online.univ-nantes.fr. This module determines the expected power of the test of the group effect for cross-sectional studies or the test of time effect for longitudinal studies given the expected values defined by the user. This study has exemplified the importance of the determination of the expected value of the variance of the latent variable. In order to help designing studies when a Rasch model is intended for the analysis and when the expected value of the variance of the latent variable is highly uncertain, a graphical option is also available in the Raschpower module. Given the expected values for the sample size in each group (*N*_0_ and *N*_1_), the group effect (*γ*) and the item difficulties (*δ*_*j*_), it provides a chart similar to Figure 3 representing the expected power as a function of a range of values of the variance of the latent variable. This chart can help to make an informed choice and may avoid insufficiently powered studies.

## Conclusions

This study emphasizes the potential strong impact of misspecifying the variance of the latent variable in power and sample size determinations for PRO cross-sectional studies comparing two groups of patients. A variance misspecification can lead to an overestimation of the power of the test of group effect at the design stage and may result in an underpowered study.

## Additional files

Additional file 1
**Misspecification of the variance of the latent variable - Power estimated with the Raschpower procedure for different values of the variance of the latent variable** $\left (\boldsymbol {\sigma }^{\text {2}}_{\boldsymbol {\theta }}\right)$ **, the number of items (** ***J*** **), the group effect (** ***γ*** **) and the sample size per group (** ***N***_**g**_ **).**

Additional file 2
**Misspecification of the item difficulties - Power estimated with the Raschpower procedure for different values of the sample size per group (** ***N***_***g***_ **), the group effect (** ***γ*** **), the variance of the item distribution** $\left (\boldsymbol {\sigma }^{\text {2}}_{\boldsymbol {\delta }_{j}}\right)$ **, the gap between the means of the two normal distributions (** ***a*** **), the variance of the latent variable** $\left (\boldsymbol {\sigma }^{\text {2}}_{\boldsymbol {\theta }}\right)$ **and the number of items (** ***J*** **).**
