# Supplementary material for: Power and sample size determination for the group comparison of patient-reported outcomes using the Rasch model: impact of a misspecification of the parameters
Source: BMC Med Res Methodol. 2015 Mar 15;15:21. doi: 10.1186/s12874-015-0011-4 (PMC4373307; doi:10.1186/s12874-015-0011-4)
Supplement: Additional file 1 — Misspecification of the variance of the latent variable - Power estimated with the Raschpower procedure for different values of the variance of the latent variable $\left (\boldsymbol {\sigma }^{\text {2}}_{\boldsymbol {\theta }}\right)$σθ2 , the number of items ( J ), the group effect ( γ ) and the sample size per group ( N g ). [file 12874_2015_11_MOESM1_ESM.docx]

Power estimated with the Raschpower procedure for different values of the variance of the latent variable ($\sigma_{\theta}^{2}$), the group effect (γ) and the sample size per group (N_g_) and the number of items J=3.

|  |  |  | $\sigma_{\theta}^{2}$ | | | | | | | | | | | | | | |
| --- | --- | --- | --- | --- | --- | --- | --- | --- | --- | --- | --- | --- | --- | --- | --- | --- | --- |
| J | N_g_ | γ | 0.25 | 0.5 | 0.75 | 1 | 1.5 | 2 | 2.5 | 3 | 3.5 | 4 | 5 | 6 | 7 | 8 | 9 |
| 3 | 50 | 0.1 | 0.058 | 0.054 | 0.051 | 0.049 | 0.046 | 0.044 | 0.042 | 0.041 | 0.040 | 0.039 | 0.037 | 0.036 | 0.036 | 0.035 | 0.034 |
| 3 | 50 | 0.2 | 0.117 | 0.104 | 0.095 | 0.088 | 0.079 | 0.072 | 0.067 | 0.064 | 0.061 | 0.058 | 0.054 | 0.052 | 0.050 | 0.048 | 0.046 |
| 3 | 50 | 0.5 | 0.482 | 0.417 | 0.367 | 0.328 | 0.275 | 0.237 | 0.211 | 0.191 | 0.175 | 0.162 | 0.143 | 0.129 | 0.118 | 0.110 | 0.104 |
| 3 | 50 | 0.8 | 0.859 | 0.793 | 0.731 | 0.677 | 0.583 | 0.511 | 0.454 | 0.408 | 0.372 | 0.343 | 0.296 | 0.262 | 0.236 | 0.216 | 0.199 |
| 3 | 100 | 0.1 | 0.078 | 0.072 | 0.067 | 0.063 | 0.058 | 0.054 | 0.051 | 0.049 | 0.047 | 0.046 | 0.044 | 0.042 | 0.041 | 0.040 | 0.039 |
| 3 | 100 | 0.2 | 0.192 | 0.167 | 0.149 | 0.136 | 0.117 | 0.105 | 0.096 | 0.089 | 0.084 | 0.079 | 0.073 | 0.068 | 0.064 | 0.061 | 0.059 |
| 3 | 100 | 0.5 | 0.773 | 0.695 | 0.630 | 0.573 | 0.486 | 0.421 | 0.373 | 0.335 | 0.304 | 0.280 | 0.242 | 0.215 | 0.195 | 0.178 | 0.165 |
| 3 | 100 | 0.8 | 0.990 | 0.975 | 0.954 | 0.927 | 0.865 | 0.803 | 0.741 | 0.687 | 0.638 | 0.594 | 0.521 | 0.465 | 0.419 | 0.382 | 0.351 |
| 3 | 200 | 0.1 | 0.117 | 0.104 | 0.095 | 0.088 | 0.079 | 0.072 | 0.067 | 0.064 | 0.061 | 0.058 | 0.054 | 0.052 | 0.050 | 0.048 | 0.046 |
| 3 | 200 | 0.2 | 0.337 | 0.289 | 0.254 | 0.229 | 0.192 | 0.168 | 0.151 | 0.137 | 0.127 | 0.119 | 0.106 | 0.097 | 0.090 | 0.085 | 0.081 |
| 3 | 200 | 0.5 | 0.969 | 0.938 | 0.900 | 0.859 | 0.777 | 0.702 | 0.638 | 0.583 | 0.535 | 0.495 | 0.430 | 0.381 | 0.342 | 0.312 | 0.287 |
| 3 | 200 | 0.8 | 1.000 | 1.000 | 0.999 | 0.998 | 0.991 | 0.978 | 0.958 | 0.933 | 0.905 | 0.875 | 0.813 | 0.754 | 0.700 | 0.651 | 0.607 |
| 3 | 300 | 0.1 | 0.155 | 0.136 | 0.122 | 0.112 | 0.098 | 0.089 | 0.082 | 0.077 | 0.072 | 0.069 | 0.064 | 0.060 | 0.057 | 0.055 | 0.053 |
| 3 | 300 | 0.2 | 0.470 | 0.405 | 0.356 | 0.319 | 0.266 | 0.230 | 0.204 | 0.185 | 0.170 | 0.157 | 0.139 | 0.125 | 0.115 | 0.107 | 0.101 |
| 3 | 300 | 0.5 | 0.997 | 0.990 | 0.978 | 0.960 | 0.915 | 0.862 | 0.808 | 0.757 | 0.709 | 0.664 | 0.589 | 0.527 | 0.477 | 0.436 | 0.401 |
| 3 | 300 | 0.8 | 1.000 | 1.000 | 1.000 | 1.000 | 1.000 | 0.998 | 0.995 | 0.989 | 0.980 | 0.968 | 0.937 | 0.900 | 0.860 | 0.820 | 0.780 |
| 3 | 500 | 0.1 | 0.229 | 0.198 | 0.176 | 0.159 | 0.136 | 0.121 | 0.110 | 0.101 | 0.095 | 0.090 | 0.081 | 0.076 | 0.071 | 0.067 | 0.064 |
| 3 | 500 | 0.2 | 0.682 | 0.602 | 0.538 | 0.485 | 0.407 | 0.351 | 0.310 | 0.279 | 0.254 | 0.234 | 0.203 | 0.181 | 0.164 | 0.151 | 0.141 |
| 3 | 500 | 0.5 | 1.000 | 1.000 | 0.999 | 0.998 | 0.990 | 0.976 | 0.955 | 0.929 | 0.899 | 0.868 | 0.805 | 0.745 | 0.690 | 0.641 | 0.598 |
| 3 | 500 | 0.8 | 1.000 | 1.000 | 1.000 | 1.000 | 1.000 | 1.000 | 1.000 | 1.000 | 0.999 | 0.998 | 0.995 | 0.987 | 0.975 | 0.960 | 0.942 |

Power estimated with the Raschpower procedure for different values of the variance of the latent variable ($\sigma_{\theta}^{2}$), the group effect (γ) and the sample size per group (N_g_) and the number of items J=5.

|  |  |  | $\sigma_{\theta}^{2}$ | | | | | | | | | | | | | | |
| --- | --- | --- | --- | --- | --- | --- | --- | --- | --- | --- | --- | --- | --- | --- | --- | --- | --- |
| J | N_g_ | γ | 0.25 | 0.5 | 0.75 | 1 | 1.5 | 2 | 2.5 | 3 | 3.5 | 4 | 5 | 6 | 7 | 8 | 9 |
| 5 | 50 | 0.1 | 0.069 | 0.062 | 0.057 | 0.054 | 0.049 | 0.046 | 0.044 | 0.042 | 0.041 | 0.040 | 0.038 | 0.037 | 0.036 | 0.036 | 0.035 |
| 5 | 50 | 0.2 | 0.155 | 0.131 | 0.115 | 0.104 | 0.089 | 0.080 | 0.073 | 0.069 | 0.065 | 0.062 | 0.057 | 0.054 | 0.052 | 0.050 | 0.048 |
| 5 | 50 | 0.5 | 0.652 | 0.550 | 0.472 | 0.414 | 0.334 | 0.282 | 0.245 | 0.219 | 0.198 | 0.182 | 0.158 | 0.141 | 0.129 | 0.119 | 0.111 |
| 5 | 50 | 0.8 | 0.962 | 0.913 | 0.853 | 0.795 | 0.686 | 0.598 | 0.528 | 0.473 | 0.428 | 0.390 | 0.334 | 0.292 | 0.262 | 0.238 | 0.218 |
| 5 | 100 | 0.1 | 0.098 | 0.086 | 0.077 | 0.071 | 0.064 | 0.058 | 0.055 | 0.052 | 0.050 | 0.048 | 0.046 | 0.044 | 0.042 | 0.041 | 0.040 |
| 5 | 100 | 0.2 | 0.266 | 0.218 | 0.187 | 0.166 | 0.138 | 0.120 | 0.108 | 0.099 | 0.092 | 0.086 | 0.078 | 0.072 | 0.068 | 0.064 | 0.062 |
| 5 | 100 | 0.5 | 0.914 | 0.838 | 0.762 | 0.694 | 0.582 | 0.499 | 0.436 | 0.388 | 0.350 | 0.319 | 0.272 | 0.239 | 0.214 | 0.196 | 0.180 |
| 5 | 100 | 0.8 | 1.000 | 0.997 | 0.989 | 0.976 | 0.933 | 0.878 | 0.819 | 0.763 | 0.710 | 0.662 | 0.581 | 0.516 | 0.464 | 0.422 | 0.386 |
| 5 | 200 | 0.1 | 0.155 | 0.131 | 0.115 | 0.104 | 0.089 | 0.080 | 0.073 | 0.069 | 0.065 | 0.062 | 0.057 | 0.054 | 0.052 | 0.050 | 0.048 |
| 5 | 200 | 0.2 | 0.472 | 0.387 | 0.329 | 0.287 | 0.232 | 0.197 | 0.173 | 0.156 | 0.142 | 0.132 | 0.116 | 0.105 | 0.097 | 0.091 | 0.086 |
| 5 | 200 | 0.5 | 0.997 | 0.986 | 0.966 | 0.937 | 0.866 | 0.790 | 0.720 | 0.659 | 0.604 | 0.558 | 0.482 | 0.425 | 0.380 | 0.344 | 0.315 |
| 5 | 200 | 0.8 | 1.000 | 1.000 | 1.000 | 1.000 | 0.998 | 0.993 | 0.982 | 0.966 | 0.945 | 0.920 | 0.865 | 0.808 | 0.753 | 0.702 | 0.656 |
| 5 | 300 | 0.1 | 0.211 | 0.175 | 0.151 | 0.135 | 0.114 | 0.100 | 0.091 | 0.084 | 0.079 | 0.074 | 0.068 | 0.063 | 0.060 | 0.057 | 0.055 |
| 5 | 300 | 0.2 | 0.639 | 0.535 | 0.459 | 0.402 | 0.324 | 0.273 | 0.238 | 0.212 | 0.192 | 0.177 | 0.154 | 0.137 | 0.125 | 0.116 | 0.108 |
| 5 | 300 | 0.5 | 1.000 | 0.999 | 0.996 | 0.990 | 0.964 | 0.924 | 0.876 | 0.827 | 0.778 | 0.732 | 0.650 | 0.582 | 0.526 | 0.479 | 0.440 |
| 5 | 300 | 0.8 | 1.000 | 1.000 | 1.000 | 1.000 | 1.000 | 1.000 | 0.999 | 0.996 | 0.992 | 0.985 | 0.963 | 0.934 | 0.899 | 0.863 | 0.825 |
| 5 | 500 | 0.1 | 0.321 | 0.262 | 0.223 | 0.197 | 0.161 | 0.139 | 0.124 | 0.113 | 0.105 | 0.098 | 0.088 | 0.081 | 0.075 | 0.071 | 0.068 |
| 5 | 500 | 0.2 | 0.848 | 0.753 | 0.669 | 0.599 | 0.493 | 0.418 | 0.364 | 0.323 | 0.291 | 0.265 | 0.227 | 0.200 | 0.180 | 0.165 | 0.153 |
| 5 | 500 | 0.5 | 1.000 | 1.000 | 1.000 | 1.000 | 0.998 | 0.992 | 0.980 | 0.963 | 0.940 | 0.915 | 0.857 | 0.799 | 0.744 | 0.693 | 0.647 |
| 5 | 500 | 0.8 | 1.000 | 1.000 | 1.000 | 1.000 | 1.000 | 1.000 | 1.000 | 1.000 | 1.000 | 1.000 | 0.998 | 0.994 | 0.987 | 0.976 | 0.962 |

Power estimated with the Raschpower procedure for different values of the variance of the latent variable ($\sigma_{\theta}^{2}$), the group effect (γ) and the sample size per group (N_g_) and the number of items J=7.

|  |  |  | $\sigma_{\theta}^{2}$ | | | | | | | | | | | | | | |
| --- | --- | --- | --- | --- | --- | --- | --- | --- | --- | --- | --- | --- | --- | --- | --- | --- | --- |
| J | N_g_ | γ | 0.25 | 0.5 | 0.75 | 1 | 1.5 | 2 | 2.5 | 3 | 3.5 | 4 | 5 | 6 | 7 | 8 | 9 |
| 7 | 50 | 0.1 | 0.077 | 0.067 | 0.061 | 0.057 | 0.051 | 0.048 | 0.045 | 0.043 | 0.042 | 0.041 | 0.039 | 0.038 | 0.037 | 0.036 | 0.035 |
| 7 | 50 | 0.2 | 0.186 | 0.150 | 0.128 | 0.114 | 0.095 | 0.084 | 0.077 | 0.072 | 0.067 | 0.064 | 0.059 | 0.055 | 0.053 | 0.051 | 0.049 |
| 7 | 50 | 0.5 | 0.755 | 0.633 | 0.537 | 0.467 | 0.370 | 0.308 | 0.265 | 0.235 | 0.212 | 0.193 | 0.166 | 0.148 | 0.134 | 0.123 | 0.115 |
| 7 | 50 | 0.8 | 0.987 | 0.954 | 0.906 | 0.849 | 0.741 | 0.645 | 0.569 | 0.506 | 0.457 | 0.417 | 0.354 | 0.310 | 0.275 | 0.249 | 0.228 |
| 7 | 100 | 0.1 | 0.114 | 0.096 | 0.084 | 0.077 | 0.067 | 0.061 | 0.057 | 0.054 | 0.051 | 0.049 | 0.047 | 0.044 | 0.043 | 0.042 | 0.040 |
| 7 | 100 | 0.2 | 0.327 | 0.257 | 0.214 | 0.186 | 0.150 | 0.129 | 0.114 | 0.104 | 0.096 | 0.090 | 0.081 | 0.075 | 0.070 | 0.066 | 0.063 |
| 7 | 100 | 0.5 | 0.964 | 0.902 | 0.830 | 0.757 | 0.636 | 0.543 | 0.472 | 0.418 | 0.375 | 0.340 | 0.289 | 0.252 | 0.226 | 0.205 | 0.188 |
| 7 | 100 | 0.8 | 1.000 | 0.999 | 0.996 | 0.989 | 0.957 | 0.910 | 0.855 | 0.800 | 0.747 | 0.697 | 0.612 | 0.543 | 0.488 | 0.442 | 0.404 |
| 7 | 200 | 0.1 | 0.187 | 0.150 | 0.128 | 0.114 | 0.096 | 0.085 | 0.077 | 0.072 | 0.067 | 0.064 | 0.059 | 0.055 | 0.053 | 0.051 | 0.049 |
| 7 | 200 | 0.2 | 0.571 | 0.456 | 0.379 | 0.326 | 0.257 | 0.215 | 0.187 | 0.166 | 0.151 | 0.139 | 0.122 | 0.110 | 0.101 | 0.094 | 0.088 |
| 7 | 200 | 0.5 | 1.000 | 0.996 | 0.985 | 0.964 | 0.904 | 0.832 | 0.762 | 0.698 | 0.641 | 0.591 | 0.510 | 0.449 | 0.400 | 0.361 | 0.330 |
| 7 | 200 | 0.8 | 1.000 | 1.000 | 1.000 | 1.000 | 0.999 | 0.997 | 0.990 | 0.977 | 0.960 | 0.938 | 0.888 | 0.833 | 0.779 | 0.728 | 0.681 |
| 7 | 300 | 0.1 | 0.258 | 0.204 | 0.172 | 0.150 | 0.123 | 0.107 | 0.096 | 0.088 | 0.082 | 0.077 | 0.070 | 0.065 | 0.062 | 0.059 | 0.056 |
| 7 | 300 | 0.2 | 0.745 | 0.619 | 0.525 | 0.455 | 0.360 | 0.299 | 0.258 | 0.228 | 0.205 | 0.188 | 0.162 | 0.144 | 0.131 | 0.120 | 0.112 |
| 7 | 300 | 0.5 | 1.000 | 1.000 | 0.999 | 0.996 | 0.979 | 0.948 | 0.906 | 0.859 | 0.811 | 0.765 | 0.681 | 0.611 | 0.552 | 0.502 | 0.461 |
| 7 | 300 | 0.8 | 1.000 | 1.000 | 1.000 | 1.000 | 1.000 | 1.000 | 0.999 | 0.998 | 0.995 | 0.990 | 0.973 | 0.948 | 0.916 | 0.882 | 0.846 |
| 7 | 500 | 0.1 | 0.394 | 0.309 | 0.257 | 0.221 | 0.177 | 0.151 | 0.133 | 0.120 | 0.110 | 0.103 | 0.092 | 0.084 | 0.078 | 0.073 | 0.070 |
| 7 | 500 | 0.2 | 0.923 | 0.832 | 0.743 | 0.665 | 0.543 | 0.457 | 0.394 | 0.348 | 0.312 | 0.283 | 0.241 | 0.211 | 0.189 | 0.172 | 0.159 |
| 7 | 500 | 0.5 | 1.000 | 1.000 | 1.000 | 1.000 | 0.999 | 0.996 | 0.988 | 0.975 | 0.956 | 0.934 | 0.881 | 0.825 | 0.770 | 0.719 | 0.671 |
| 7 | 500 | 0.8 | 1.000 | 1.000 | 1.000 | 1.000 | 1.000 | 1.000 | 1.000 | 1.000 | 1.000 | 1.000 | 0.999 | 0.996 | 0.991 | 0.982 | 0.970 |

Power estimated with the Raschpower procedure for different values of the variance of the latent variable ($\sigma_{\theta}^{2}$), the group effect (γ) and the sample size per group (N_g_) and the number of items J=9.

|  |  |  | $\sigma_{\theta}^{2}$ | | | | | | | | | | | | | | |
| --- | --- | --- | --- | --- | --- | --- | --- | --- | --- | --- | --- | --- | --- | --- | --- | --- | --- |
| J | N | γ | 0.25 | 0.5 | 0.75 | 1 | 1.5 | 2 | 2.5 | 3 | 3.5 | 4 | 5 | 6 | 7 | 8 | 9 |
| 9 | 50 | 0.1 | 0.084 | 0.071 | 0.064 | 0.059 | 0.052 | 0.049 | 0.046 | 0.044 | 0.043 | 0.041 | 0.039 | 0.038 | 0.037 | 0.036 | 0.036 |
| 9 | 50 | 0.2 | 0.209 | 0.164 | 0.138 | 0.121 | 0.100 | 0.088 | 0.079 | 0.073 | 0.069 | 0.065 | 0.060 | 0.056 | 0.053 | 0.051 | 0.049 |
| 9 | 50 | 0.5 | 0.798 | 0.682 | 0.579 | 0.501 | 0.394 | 0.325 | 0.279 | 0.245 | 0.220 | 0.200 | 0.172 | 0.152 | 0.137 | 0.126 | 0.118 |
| 9 | 50 | 0.8 | 0.991 | 0.970 | 0.929 | 0.877 | 0.770 | 0.674 | 0.592 | 0.529 | 0.477 | 0.433 | 0.367 | 0.320 | 0.284 | 0.257 | 0.234 |
| 9 | 100 | 0.1 | 0.128 | 0.103 | 0.089 | 0.081 | 0.069 | 0.063 | 0.058 | 0.055 | 0.052 | 0.050 | 0.047 | 0.045 | 0.043 | 0.042 | 0.041 |
| 9 | 100 | 0.2 | 0.375 | 0.285 | 0.233 | 0.200 | 0.159 | 0.135 | 0.119 | 0.108 | 0.099 | 0.093 | 0.083 | 0.076 | 0.071 | 0.067 | 0.064 |
| 9 | 100 | 0.5 | 0.981 | 0.934 | 0.867 | 0.795 | 0.668 | 0.570 | 0.495 | 0.437 | 0.391 | 0.355 | 0.299 | 0.261 | 0.232 | 0.210 | 0.193 |
| 9 | 100 | 0.8 | 1.000 | 1.000 | 0.998 | 0.993 | 0.969 | 0.926 | 0.875 | 0.820 | 0.767 | 0.717 | 0.631 | 0.560 | 0.503 | 0.455 | 0.416 |
| 9 | 200 | 0.1 | 0.213 | 0.165 | 0.138 | 0.121 | 0.100 | 0.088 | 0.079 | 0.073 | 0.069 | 0.065 | 0.060 | 0.056 | 0.053 | 0.051 | 0.049 |
| 9 | 200 | 0.2 | 0.643 | 0.505 | 0.415 | 0.352 | 0.274 | 0.227 | 0.196 | 0.173 | 0.157 | 0.144 | 0.126 | 0.113 | 0.103 | 0.096 | 0.090 |
| 9 | 200 | 0.5 | 1.000 | 0.998 | 0.992 | 0.977 | 0.924 | 0.856 | 0.786 | 0.721 | 0.663 | 0.612 | 0.528 | 0.463 | 0.412 | 0.372 | 0.340 |
| 9 | 200 | 0.8 | 1.000 | 1.000 | 1.000 | 1.000 | 1.000 | 0.998 | 0.993 | 0.983 | 0.968 | 0.948 | 0.901 | 0.847 | 0.794 | 0.743 | 0.696 |
| 9 | 300 | 0.1 | 0.297 | 0.226 | 0.187 | 0.161 | 0.130 | 0.112 | 0.099 | 0.091 | 0.084 | 0.079 | 0.072 | 0.066 | 0.063 | 0.059 | 0.057 |
| 9 | 300 | 0.2 | 0.814 | 0.677 | 0.570 | 0.491 | 0.384 | 0.317 | 0.271 | 0.239 | 0.214 | 0.195 | 0.167 | 0.148 | 0.134 | 0.123 | 0.115 |
| 9 | 300 | 0.5 | 1.000 | 1.000 | 1.000 | 0.998 | 0.986 | 0.960 | 0.921 | 0.877 | 0.831 | 0.785 | 0.701 | 0.628 | 0.568 | 0.517 | 0.474 |
| 9 | 300 | 0.8 | 1.000 | 1.000 | 1.000 | 1.000 | 1.000 | 1.000 | 1.000 | 0.999 | 0.997 | 0.993 | 0.978 | 0.955 | 0.926 | 0.893 | 0.857 |
| 9 | 500 | 0.1 | 0.453 | 0.345 | 0.281 | 0.239 | 0.188 | 0.158 | 0.138 | 0.124 | 0.114 | 0.106 | 0.094 | 0.085 | 0.079 | 0.074 | 0.071 |
| 9 | 500 | 0.2 | 0.958 | 0.878 | 0.788 | 0.707 | 0.575 | 0.482 | 0.415 | 0.364 | 0.325 | 0.295 | 0.250 | 0.218 | 0.195 | 0.177 | 0.163 |
| 9 | 500 | 0.5 | 1.000 | 1.000 | 1.000 | 1.000 | 1.000 | 0.998 | 0.992 | 0.981 | 0.965 | 0.944 | 0.894 | 0.840 | 0.785 | 0.734 | 0.686 |
| 9 | 500 | 0.8 | 1.000 | 1.000 | 1.000 | 1.000 | 1.000 | 1.000 | 1.000 | 1.000 | 1.000 | 1.000 | 0.999 | 0.997 | 0.993 | 0.985 | 0.974 |

Power estimated with the Raschpower procedure for different values of the variance of the latent variable ($\sigma_{\theta}^{2}$), the group effect (γ) and the sample size per group (N_g_) and the number of items J=11.

|  |  |  | $\sigma_{\theta}^{2}$ | | | | | | | | | | | | | | |
| --- | --- | --- | --- | --- | --- | --- | --- | --- | --- | --- | --- | --- | --- | --- | --- | --- | --- |
| J | N | γ | 0.25 | 0.5 | 0.75 | 1 | 1.5 | 2 | 2.5 | 3 | 3.5 | 4 | 5 | 6 | 7 | 8 | 9 |
| 11 | 50 | 0.1 | 0.089 | 0.074 | 0.065 | 0.060 | 0.053 | 0.049 | 0.046 | 0.044 | 0.043 | 0.042 | 0.040 | 0.038 | 0.037 | 0.036 | 0.036 |
| 11 | 50 | 0.2 | 0.225 | 0.173 | 0.144 | 0.125 | 0.103 | 0.089 | 0.081 | 0.074 | 0.070 | 0.066 | 0.061 | 0.057 | 0.054 | 0.052 | 0.050 |
| 11 | 50 | 0.5 | 0.790 | 0.705 | 0.603 | 0.520 | 0.409 | 0.337 | 0.287 | 0.252 | 0.226 | 0.206 | 0.175 | 0.155 | 0.140 | 0.128 | 0.119 |
| 11 | 50 | 0.8 | 0.991 | 0.969 | 0.938 | 0.892 | 0.786 | 0.690 | 0.610 | 0.542 | 0.488 | 0.443 | 0.375 | 0.327 | 0.290 | 0.262 | 0.238 |
| 11 | 100 | 0.1 | 0.138 | 0.109 | 0.093 | 0.083 | 0.071 | 0.064 | 0.059 | 0.056 | 0.053 | 0.051 | 0.048 | 0.045 | 0.044 | 0.042 | 0.041 |
| 11 | 100 | 0.2 | 0.408 | 0.305 | 0.246 | 0.209 | 0.164 | 0.138 | 0.122 | 0.110 | 0.101 | 0.094 | 0.084 | 0.077 | 0.072 | 0.068 | 0.065 |
| 11 | 100 | 0.5 | 0.986 | 0.948 | 0.887 | 0.818 | 0.689 | 0.588 | 0.510 | 0.449 | 0.401 | 0.364 | 0.306 | 0.267 | 0.237 | 0.214 | 0.197 |
| 11 | 100 | 0.8 | 1.000 | 1.000 | 0.999 | 0.995 | 0.975 | 0.936 | 0.887 | 0.834 | 0.781 | 0.732 | 0.643 | 0.571 | 0.512 | 0.464 | 0.424 |
| 11 | 200 | 0.1 | 0.235 | 0.177 | 0.146 | 0.126 | 0.103 | 0.090 | 0.081 | 0.075 | 0.070 | 0.066 | 0.061 | 0.057 | 0.054 | 0.052 | 0.050 |
| 11 | 200 | 0.2 | 0.693 | 0.541 | 0.440 | 0.371 | 0.286 | 0.235 | 0.202 | 0.178 | 0.161 | 0.147 | 0.128 | 0.115 | 0.105 | 0.097 | 0.091 |
| 11 | 200 | 0.5 | 1.000 | 0.999 | 0.995 | 0.983 | 0.936 | 0.871 | 0.802 | 0.737 | 0.678 | 0.626 | 0.539 | 0.473 | 0.421 | 0.380 | 0.346 |
| 11 | 200 | 0.8 | 1.000 | 1.000 | 1.000 | 1.000 | 1.000 | 0.999 | 0.994 | 0.986 | 0.972 | 0.954 | 0.909 | 0.857 | 0.804 | 0.753 | 0.706 |
| 11 | 300 | 0.1 | 0.329 | 0.244 | 0.198 | 0.169 | 0.134 | 0.115 | 0.102 | 0.093 | 0.086 | 0.081 | 0.073 | 0.067 | 0.063 | 0.060 | 0.057 |
| 11 | 300 | 0.2 | 0.857 | 0.716 | 0.602 | 0.517 | 0.401 | 0.329 | 0.280 | 0.246 | 0.220 | 0.200 | 0.171 | 0.151 | 0.136 | 0.125 | 0.116 |
| 11 | 300 | 0.5 | 1.000 | 1.000 | 1.000 | 0.999 | 0.989 | 0.966 | 0.931 | 0.889 | 0.843 | 0.798 | 0.713 | 0.640 | 0.578 | 0.526 | 0.483 |
| 11 | 300 | 0.8 | 1.000 | 1.000 | 1.000 | 1.000 | 1.000 | 1.000 | 1.000 | 0.999 | 0.997 | 0.994 | 0.981 | 0.960 | 0.932 | 0.900 | 0.865 |
| 11 | 500 | 0.1 | 0.500 | 0.373 | 0.300 | 0.253 | 0.196 | 0.163 | 0.142 | 0.128 | 0.116 | 0.108 | 0.095 | 0.087 | 0.080 | 0.075 | 0.071 |
| 11 | 500 | 0.2 | 0.975 | 0.905 | 0.818 | 0.734 | 0.598 | 0.500 | 0.429 | 0.376 | 0.335 | 0.303 | 0.256 | 0.223 | 0.199 | 0.181 | 0.166 |
| 11 | 500 | 0.5 | 1.000 | 1.000 | 1.000 | 1.000 | 1.000 | 0.998 | 0.994 | 0.984 | 0.969 | 0.950 | 0.903 | 0.849 | 0.796 | 0.744 | 0.696 |
| 11 | 500 | 0.8 | 1.000 | 1.000 | 1.000 | 1.000 | 1.000 | 1.000 | 1.000 | 1.000 | 1.000 | 1.000 | 0.999 | 0.998 | 0.994 | 0.987 | 0.977 |

Power estimated with the Raschpower procedure for different values of the variance of the latent variable ($\sigma_{\theta}^{2}$), the group effect (γ) and the sample size per group (N_g_) and the number of items J=13.

|  |  |  | $\sigma_{\theta}^{2}$ | | | | | | | | | | | | | | |
| --- | --- | --- | --- | --- | --- | --- | --- | --- | --- | --- | --- | --- | --- | --- | --- | --- | --- |
| J | N | γ | 0.25 | 0.5 | 0.75 | 1 | 1.5 | 2 | 2.5 | 3 | 3.5 | 4 | 5 | 6 | 7 | 8 | 9 |
| 13 | 50 | 0.1 | 0.092 | 0.075 | 0.066 | 0.061 | 0.054 | 0.050 | 0.047 | 0.045 | 0.043 | 0.042 | 0.040 | 0.038 | 0.037 | 0.036 | 0.036 |
| 13 | 50 | 0.2 | 0.228 | 0.179 | 0.147 | 0.127 | 0.104 | 0.090 | 0.082 | 0.075 | 0.071 | 0.067 | 0.061 | 0.057 | 0.054 | 0.052 | 0.050 |
| 13 | 50 | 0.5 | 0.784 | 0.699 | 0.611 | 0.531 | 0.416 | 0.343 | 0.293 | 0.257 | 0.229 | 0.208 | 0.178 | 0.157 | 0.142 | 0.130 | 0.120 |
| 13 | 50 | 0.8 | 0.990 | 0.966 | 0.938 | 0.893 | 0.794 | 0.700 | 0.617 | 0.550 | 0.496 | 0.451 | 0.382 | 0.332 | 0.294 | 0.265 | 0.242 |
| 13 | 100 | 0.1 | 0.146 | 0.112 | 0.095 | 0.085 | 0.072 | 0.065 | 0.060 | 0.056 | 0.053 | 0.051 | 0.048 | 0.046 | 0.044 | 0.042 | 0.041 |
| 13 | 100 | 0.2 | 0.425 | 0.319 | 0.255 | 0.215 | 0.168 | 0.141 | 0.124 | 0.112 | 0.103 | 0.095 | 0.085 | 0.078 | 0.072 | 0.068 | 0.065 |
| 13 | 100 | 0.5 | 0.983 | 0.953 | 0.897 | 0.830 | 0.702 | 0.600 | 0.520 | 0.458 | 0.409 | 0.370 | 0.312 | 0.271 | 0.241 | 0.217 | 0.199 |
| 13 | 100 | 0.8 | 1.000 | 1.000 | 0.999 | 0.996 | 0.978 | 0.942 | 0.894 | 0.842 | 0.791 | 0.740 | 0.652 | 0.578 | 0.519 | 0.471 | 0.430 |
| 13 | 200 | 0.1 | 0.251 | 0.185 | 0.151 | 0.130 | 0.105 | 0.091 | 0.082 | 0.076 | 0.071 | 0.067 | 0.061 | 0.057 | 0.054 | 0.052 | 0.050 |
| 13 | 200 | 0.2 | 0.725 | 0.565 | 0.457 | 0.384 | 0.294 | 0.241 | 0.206 | 0.182 | 0.164 | 0.150 | 0.130 | 0.116 | 0.106 | 0.098 | 0.092 |
| 13 | 200 | 0.5 | 1.000 | 0.999 | 0.996 | 0.986 | 0.943 | 0.881 | 0.813 | 0.748 | 0.688 | 0.636 | 0.548 | 0.480 | 0.427 | 0.385 | 0.351 |
| 13 | 200 | 0.8 | 1.000 | 1.000 | 1.000 | 1.000 | 1.000 | 0.999 | 0.995 | 0.987 | 0.975 | 0.958 | 0.914 | 0.864 | 0.811 | 0.761 | 0.713 |
| 13 | 300 | 0.1 | 0.353 | 0.256 | 0.205 | 0.174 | 0.138 | 0.117 | 0.104 | 0.094 | 0.087 | 0.082 | 0.074 | 0.068 | 0.064 | 0.060 | 0.058 |
| 13 | 300 | 0.2 | 0.882 | 0.741 | 0.623 | 0.534 | 0.413 | 0.338 | 0.287 | 0.251 | 0.224 | 0.203 | 0.174 | 0.153 | 0.138 | 0.127 | 0.118 |
| 13 | 300 | 0.5 | 1.000 | 1.000 | 1.000 | 0.999 | 0.991 | 0.970 | 0.937 | 0.896 | 0.852 | 0.807 | 0.722 | 0.649 | 0.586 | 0.533 | 0.489 |
| 13 | 300 | 0.8 | 1.000 | 1.000 | 1.000 | 1.000 | 1.000 | 1.000 | 1.000 | 0.999 | 0.998 | 0.995 | 0.983 | 0.963 | 0.936 | 0.905 | 0.871 |
| 13 | 500 | 0.1 | 0.536 | 0.394 | 0.313 | 0.262 | 0.202 | 0.167 | 0.145 | 0.130 | 0.118 | 0.109 | 0.097 | 0.088 | 0.081 | 0.076 | 0.072 |
| 13 | 500 | 0.2 | 0.983 | 0.922 | 0.838 | 0.754 | 0.614 | 0.512 | 0.439 | 0.384 | 0.342 | 0.309 | 0.260 | 0.227 | 0.202 | 0.183 | 0.168 |
| 13 | 500 | 0.5 | 1.000 | 1.000 | 1.000 | 1.000 | 1.000 | 0.999 | 0.995 | 0.986 | 0.973 | 0.955 | 0.909 | 0.856 | 0.803 | 0.751 | 0.704 |
| 13 | 500 | 0.8 | 1.000 | 1.000 | 1.000 | 1.000 | 1.000 | 1.000 | 1.000 | 1.000 | 1.000 | 1.000 | 1.000 | 0.998 | 0.994 | 0.988 | 0.979 |

Power estimated with the Raschpower procedure for different values of the variance of the latent variable ($\sigma_{\theta}^{2}$), the group effect (γ) and the sample size per group (N_g_) and the number of items J=15.

|  |  |  | $\sigma_{\theta}^{2}$ | | | | | | | | | | | | | | |
| --- | --- | --- | --- | --- | --- | --- | --- | --- | --- | --- | --- | --- | --- | --- | --- | --- | --- |
| J | N | γ | 0.25 | 0.5 | 0.75 | 1 | 1.5 | 2 | 2.5 | 3 | 3.5 | 4 | 5 | 6 | 7 | 8 | 9 |
| 15 | 50 | 0.1 | 0.094 | 0.077 | 0.067 | 0.061 | 0.054 | 0.050 | 0.047 | 0.045 | 0.043 | 0.042 | 0.040 | 0.039 | 0.037 | 0.037 | 0.036 |
| 15 | 50 | 0.2 | 0.228 | 0.181 | 0.149 | 0.129 | 0.105 | 0.091 | 0.082 | 0.076 | 0.071 | 0.067 | 0.061 | 0.058 | 0.054 | 0.052 | 0.050 |
| 15 | 50 | 0.5 | 0.768 | 0.695 | 0.607 | 0.532 | 0.420 | 0.346 | 0.296 | 0.259 | 0.232 | 0.210 | 0.179 | 0.158 | 0.143 | 0.131 | 0.122 |
| 15 | 50 | 0.8 | 0.989 | 0.962 | 0.932 | 0.895 | 0.796 | 0.703 | 0.623 | 0.557 | 0.501 | 0.455 | 0.385 | 0.335 | 0.297 | 0.268 | 0.244 |
| 15 | 100 | 0.1 | 0.150 | 0.115 | 0.097 | 0.086 | 0.073 | 0.065 | 0.060 | 0.056 | 0.054 | 0.051 | 0.048 | 0.046 | 0.044 | 0.042 | 0.041 |
| 15 | 100 | 0.2 | 0.428 | 0.325 | 0.260 | 0.218 | 0.170 | 0.143 | 0.125 | 0.113 | 0.104 | 0.096 | 0.086 | 0.078 | 0.073 | 0.069 | 0.065 |
| 15 | 100 | 0.5 | 0.981 | 0.950 | 0.900 | 0.834 | 0.710 | 0.605 | 0.526 | 0.463 | 0.414 | 0.375 | 0.316 | 0.274 | 0.243 | 0.219 | 0.201 |
| 15 | 100 | 0.8 | 1.000 | 1.000 | 0.999 | 0.996 | 0.979 | 0.945 | 0.899 | 0.847 | 0.795 | 0.746 | 0.658 | 0.584 | 0.525 | 0.475 | 0.434 |
| 15 | 200 | 0.1 | 0.263 | 0.190 | 0.154 | 0.132 | 0.107 | 0.092 | 0.083 | 0.076 | 0.071 | 0.067 | 0.062 | 0.058 | 0.055 | 0.052 | 0.050 |
| 15 | 200 | 0.2 | 0.737 | 0.578 | 0.467 | 0.392 | 0.299 | 0.245 | 0.209 | 0.184 | 0.166 | 0.152 | 0.131 | 0.117 | 0.107 | 0.099 | 0.093 |
| 15 | 200 | 0.5 | 1.000 | 1.000 | 0.996 | 0.987 | 0.947 | 0.887 | 0.820 | 0.755 | 0.695 | 0.642 | 0.554 | 0.486 | 0.432 | 0.390 | 0.355 |
| 15 | 200 | 0.8 | 1.000 | 1.000 | 1.000 | 1.000 | 1.000 | 0.999 | 0.996 | 0.989 | 0.977 | 0.961 | 0.918 | 0.868 | 0.817 | 0.766 | 0.719 |
| 15 | 300 | 0.1 | 0.371 | 0.265 | 0.211 | 0.178 | 0.140 | 0.119 | 0.105 | 0.095 | 0.088 | 0.082 | 0.074 | 0.068 | 0.064 | 0.061 | 0.058 |
| 15 | 300 | 0.2 | 0.895 | 0.757 | 0.637 | 0.545 | 0.420 | 0.344 | 0.292 | 0.255 | 0.227 | 0.206 | 0.176 | 0.155 | 0.139 | 0.128 | 0.119 |
| 15 | 300 | 0.5 | 1.000 | 1.000 | 1.000 | 0.999 | 0.993 | 0.973 | 0.941 | 0.901 | 0.858 | 0.813 | 0.729 | 0.655 | 0.592 | 0.539 | 0.494 |
| 15 | 300 | 0.8 | 1.000 | 1.000 | 1.000 | 1.000 | 1.000 | 1.000 | 1.000 | 0.999 | 0.998 | 0.995 | 0.984 | 0.965 | 0.939 | 0.908 | 0.875 |
| 15 | 500 | 0.1 | 0.562 | 0.408 | 0.322 | 0.269 | 0.206 | 0.170 | 0.147 | 0.131 | 0.120 | 0.111 | 0.097 | 0.088 | 0.082 | 0.077 | 0.072 |
| 15 | 500 | 0.2 | 0.987 | 0.932 | 0.850 | 0.766 | 0.625 | 0.521 | 0.446 | 0.390 | 0.347 | 0.313 | 0.264 | 0.229 | 0.204 | 0.185 | 0.170 |
| 15 | 500 | 0.5 | 1.000 | 1.000 | 1.000 | 1.000 | 1.000 | 0.999 | 0.995 | 0.987 | 0.975 | 0.957 | 0.913 | 0.861 | 0.808 | 0.757 | 0.709 |
| 15 | 500 | 0.8 | 1.000 | 1.000 | 1.000 | 1.000 | 1.000 | 1.000 | 1.000 | 1.000 | 1.000 | 1.000 | 1.000 | 0.998 | 0.995 | 0.989 | 0.980 |
